# Large iliofemoral aneurysm harboring intramural fibrin-associated B-cell lymphoma

**DOI:** 10.1016/j.jvscit.2025.101810

**Published:** 2025-04-21

**Authors:** Christine L. Zickler, Jeffrey T. Bunning, Mikell Jarratt, Amber Johnson, Robert W. Zickler, Paul V. Kochupura

**Affiliations:** a Herbert Wertheim College of Medicine, Florida International University, Miami, FL; b Department of Pathology, CaroMont Regional Medical Center, Gastonia, NC; c Department of Pulmonology, Gastonia Medical Specialty Clinic, Gastonia, NC; d Department of Surgery, Section of Vascular Surgery, CaroMont Regional Medical Center, Gastonia, NC

**Keywords:** Aneurysm, Arterial, B-cell lymphoma, Fibrin, Mural thrombus

## Abstract

Fibrin-associated large B-cell lymphoma (FA-LBCL) is an extremely rare, non-mass forming lymphoproliferative disorder associated with Epstein-Barr virus (EBV). It is typically discovered incidentally on histologic examination of tissue removed for unrelated reasons. The following case report presents only the second reported case in the literature of FA-LBCL found proliferating within the mural thrombus of an aneurysm.

Fibrin-associated large B-cell lymphoma (FA-LBCL) is a rare, non-mass-forming lymphoproliferative disorder linked to Epstein-Barr virus (EBV). Unlike traditional lymphomas, FA-LBCL is usually discovered incidentally during histopathologic analysis of tissue removed for unrelated reasons, as it does not cause symptoms.^1^^,^^2^ The unique pathology of FA-LBCL is its confinement to fibrin-rich environments, without invasion into adjacent tissues. The literature has documented FA-LBCL in various settings, such as cardiac myxoma, cyst wall, thyroid hyperplastic nodule, metallic knee implant, and breast implant.^2^, ^3^, ^4^, ^5^, ^6^ However, FA-LBCL within a native arterial thrombus is exceedingly uncommon, with only one prior case documented to date.

This case report presents an 85-year-old man who underwent elective vascular surgery for a large iliofemoral arterial aneurysm, during which FA-LBCL was unexpectedly identified within the mural thrombus on histologic examination. It highlights the diagnostic challenges and unique management considerations associated with this rare and incidental malignancy, contributing to the limited understanding of FA-LBCL in vascular thrombus and its implications for patient outcomes and treatment strategies. The patient has given consent for the publication of this case report.

## Case report

**Fig 1:**
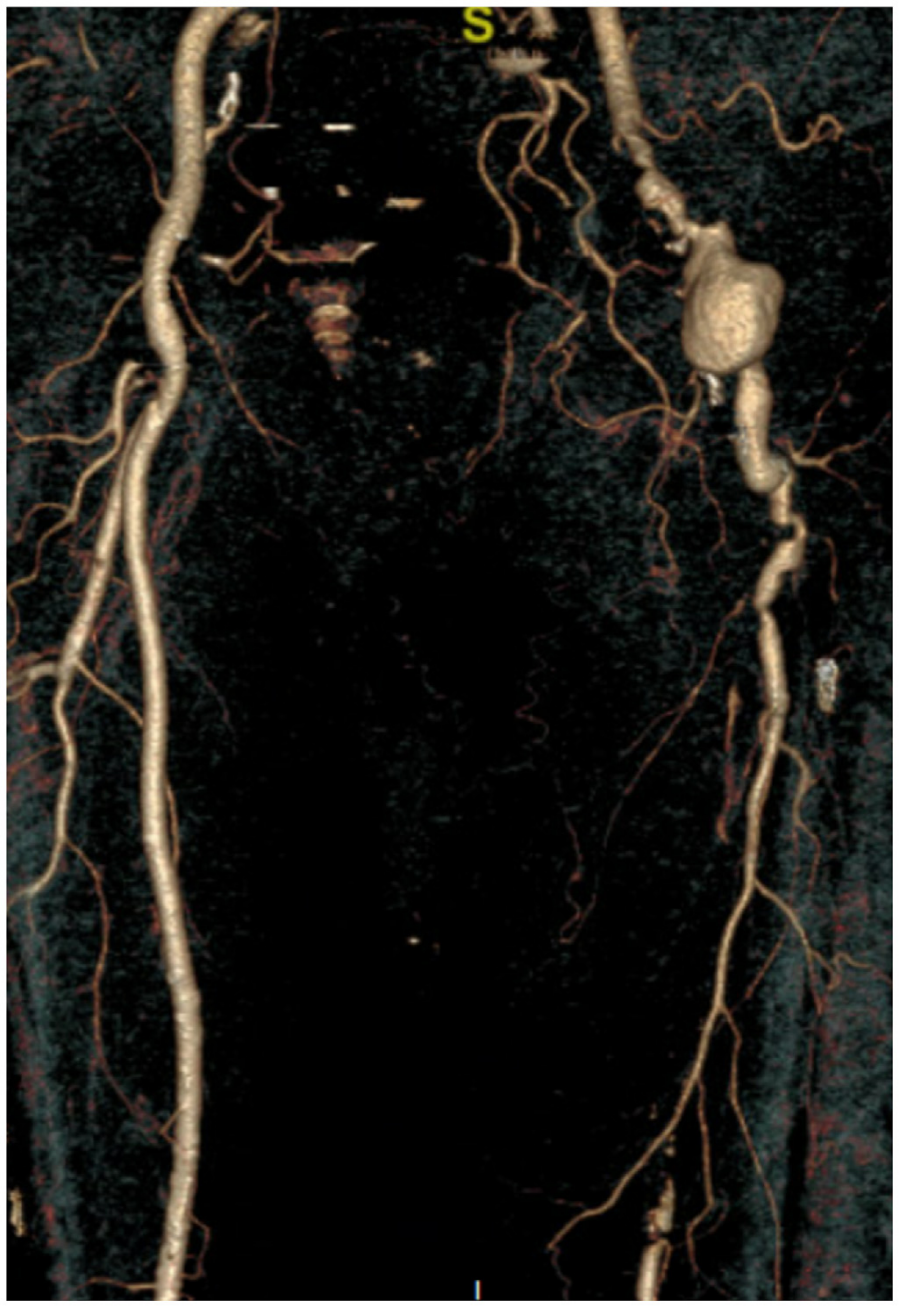
Left iliofemoral arterial aneurysm.

**Fig 2:**
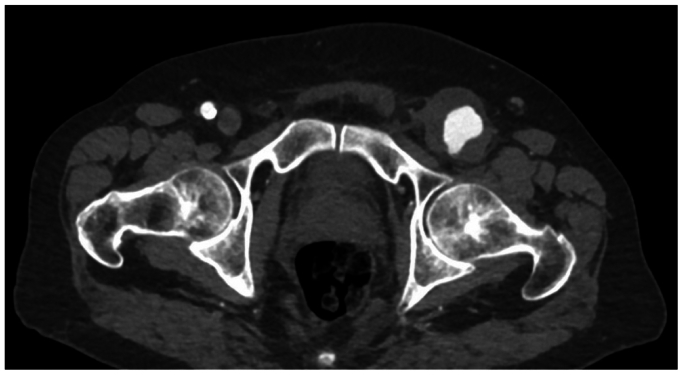
Mural thrombus in the left femoral aneurysm.

**Fig 3:**
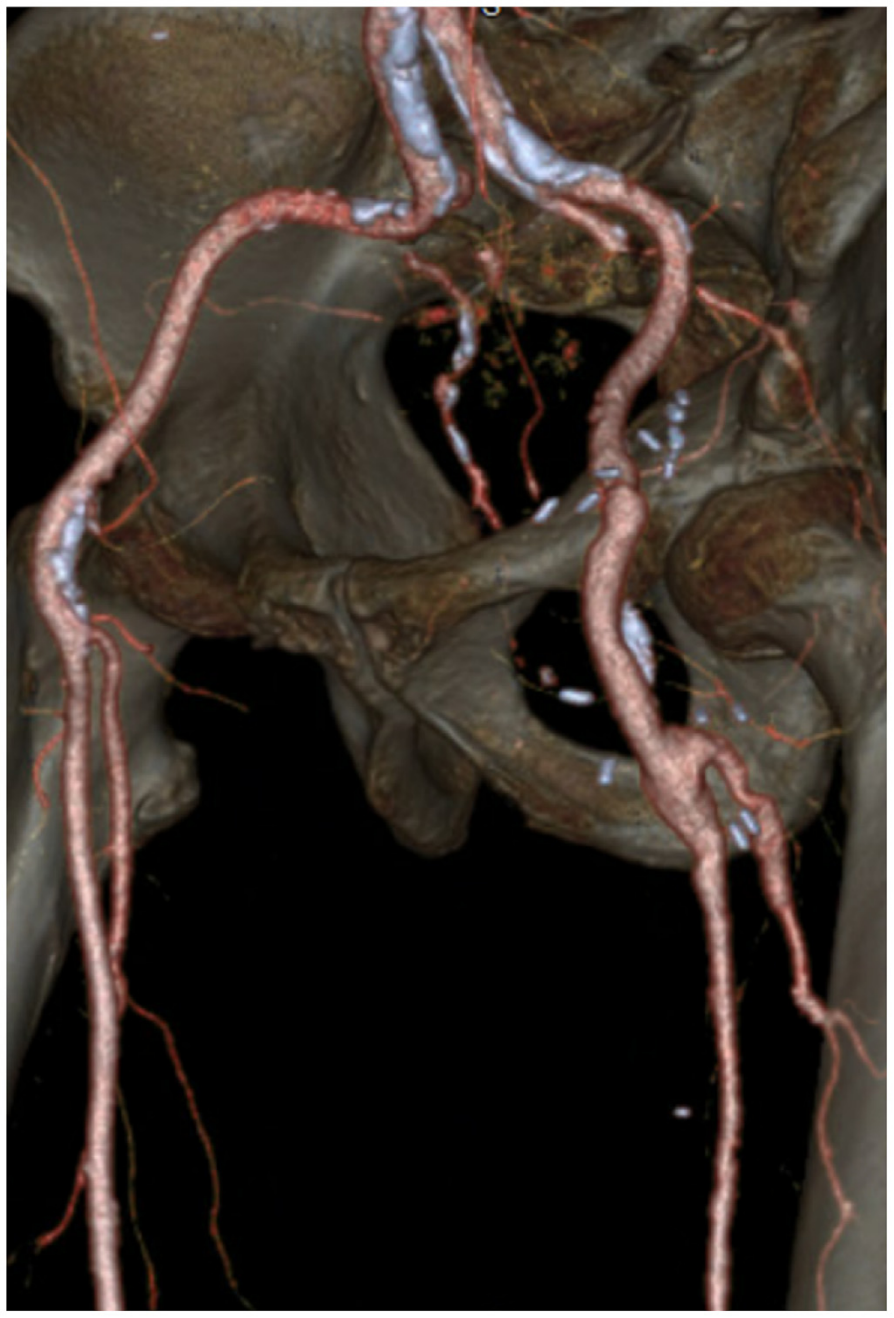
Post repair of iliofemoral aneurysm with interposition grafting and left superficial femoral artery thrombectomy.

An 85-year-old man with past medical history significant for prior T-cell large granular lymphocytic expansion, psoriasis, obstructive sleep apnea, and stage 3 chronic kidney disease underwent elective surgery for a large left iliofemoral arterial aneurysm after presenting with left leg pain. Preoperative computed tomography (CT) imaging revealed a 55 mm × 40 mm left iliofemoral aneurysm extending into the profunda femoris artery (12 mm). The left superficial femoral artery was occluded at its origin but reconstituted near the adductor canal. The popliteal artery was patent. The tibial arteries had severe atherosclerotic disease (Fig 1). There was significant mural thrombus in the aneurysmal sac (Fig 2). There was no evidence of any other aneurysms on CT angiography (CTA) of the abdomen and pelvis with bilateral lower extremity runoff and CT chest without contrast. The procedure involved open repair of the left external iliac and common femoral artery aneurysm with placement of an interposition graft and left superficial femoral artery thrombectomy (Fig 3).

**Fig 4:**
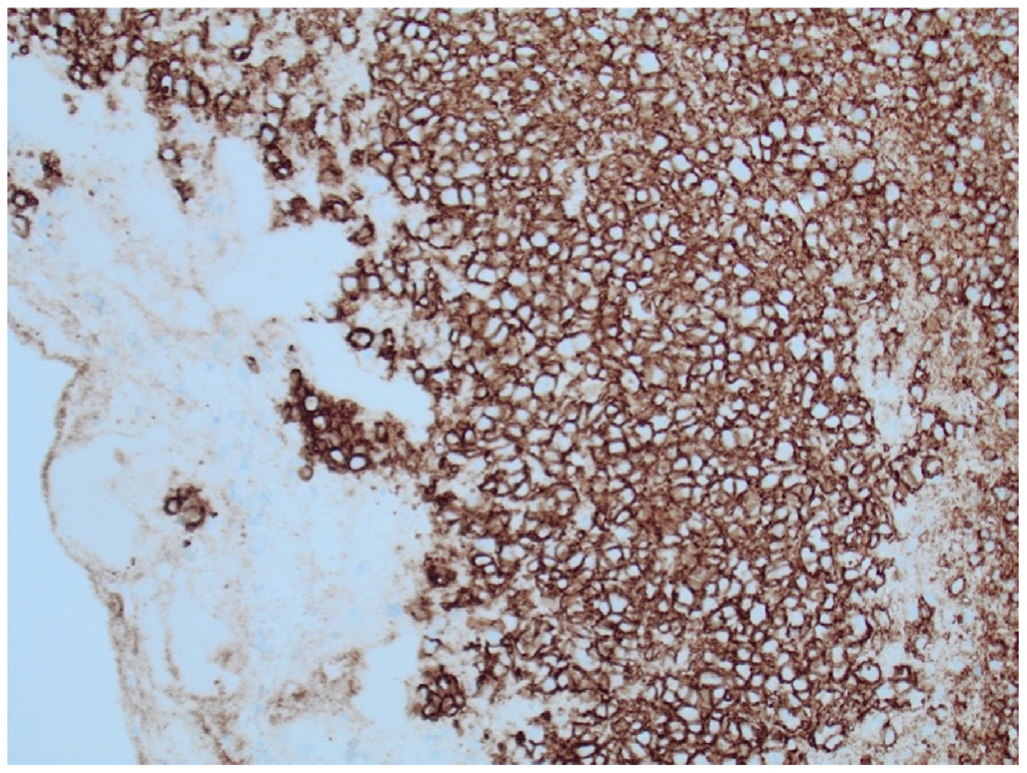
Fibrin-associated large B-cell lymphoma (FA-LBCL) within a portion of the mural thrombus. CD20 immunohistochemistry confirms B-cell lymphoma.

Following the procedure, histopathologic examination of the excised tissue revealed FA-LBCL within a portion of the mural thrombus (Fig 4). There was no involvement of the arterial wall or adjacent tissue. Additional samples of the resected aneurysmal wall tested negative for lymphoma. Imaging studies, including CT chest and CTA of the abdomen and pelvis, showed no evidence of lymphadenopathy. EBV in-situ stain was positive. Positron emission tomography scan showed small, indeterminate hypermetabolic lymph nodes in the right neck and left axilla, along with areas of elevated metabolic activity in the descending and sigmoid colon, suggestive of inflammatory changes due to marked diverticulosis. Bone marrow biopsy revealed mild hypercellular marrow with maturing trilineage hematopoiesis and scattered lymphoid aggregates, with no morphologic or immunologic evidence of lymphoma. Flow cytometry was negative for myeloid and lymphoid immunophenotypic abnormalities. Analysis of the T-cell receptor gamma gene showed a clonal population emerging from a polyclonal background. A clonal population of T-cell receptor beta was identified.

The patient also had a history of T-cell large granular lymphocyte expansion, first noted 8 years prior to the current FA-LBCL diagnosis. At that time, flow cytometry of the bone marrow aspirate did not show any overt myeloid or lymphoid immunophenotypic abnormalities. T-cell receptor gene rearrangement showed a clonal population from a polyclonal background. Differential diagnoses included reactive process (in the setting of psoriasis) and malignant clone.

Once the patient’s wound healed, hematology/oncology started him on rituximab therapy. He was also referred to radiation oncology for radiation therapy to the involved site. The patient ultimately received this care at another facility. As of the most recent available documentation, he had started rituximab treatment but had not yet undergone radiation therapy, and no follow-up imaging was available for review.

## Discussion

FA-LBCL is exceptionally rare, with its behavior exhibiting significant variability. In the literature, most cases of FA-LBCL are found incidentally because they do not produce symptoms. All cases were diagnosed postoperatively.^2^, ^3^, ^4^, ^5^, ^6^ Due to its rarity, there are no established guidelines for the management of FA-LBCL. Management options include observation, treatment of EBV infection, and in some cases, chemotherapy and radiation.

FA-LBCL in a mural thrombus presents additional challenges because it is so rare. There is only one other reported case of FA-LBCL in a mural thrombus in the literature. The case report by Habib et al details this discovery in a 77-year-old man who underwent surgery for aortoiliac occlusive disease with an open aorto-biiliac bypass. The authors describe “suspicious inflammatory changes” around the aorta. Mural thrombus of the infrarenal aorta and bilaterial renal arteries was removed and analyzed. The pathology reports detailed a B-cell lymphoma with BCL2, MYC, CD30, and EBV positivity. This patient’s care was complicated by acute tubular necrosis requiring hemodialysis. The patient died from renal complications, and he did not undergo treatment for his FA-LBCL.^7^

In addition to FA-LBCL, other hematogenous-based malignancies have been reported within arterial mural thrombi, although such occurrences remain exceedingly rare. These include intravascular large B-cell lymphoma, which can mimic thromboembolic disease and has been identified within arterial thrombi, such as in the aorta and femoral artery.^8^ Similarly, metastatic solid tumors like renal cell carcinoma and sarcomatoid carcinoma have been found within thrombi of the aortic arch and aortic grafts, presenting as arterial occlusions or embolic events.^9^^,^^10^ Histopathologic examination remains essential for diagnosis, as these malignancies are often discovered incidentally during thrombectomy or aneurysm repair.^8^, ^9^, ^10^ The identification of malignant cells within mural thrombi highlights the importance of routine pathologic analysis in cases of atypical thrombus presentation, particularly in patients with a known history or risk of malignancy.

The natural history of FA-LBCL is characterized by its indolent, noninvasive course, with cases presenting incidentally and showing no evidence of local tissue infiltration or systemic spread. Surgical excision is often curative, and long-term follow-up studies report excellent outcomes with minimal risk of recurrence or progression, particularly when the lesion is completely resected.^11^

There are no specific radiographic findings that suggest the presence of FA-LBCL preoperatively; it is almost always discovered incidentally on histopathology, as was the case here.^1^^,^^11^ Standard imaging modalities used for aneurysm evaluation, including CTA, are not capable of distinguishing this process from typical mural thrombus. Although a positron emission tomography-CT scan may be useful postoperatively to monitor for residual or recurrent disease, it is not part of standard preoperative workup.^11^ Importantly, if an endovascular approach had been used to repair this aneurysm, the diagnosis of FA-LBCL would have been missed entirely. However, given the extreme rarity of this entity, this should not alter clinical decision-making regarding endovascular vs open surgical repair of aneurysms.

Although the significance of the patient’s prior T-cell granular lymphocyte expansion remains unclear, rare cases of co-occurring T-cell large granular lymphocyte proliferations and EBV-positive B-cell lymphomas have been reported, suggesting a potential shared immune dysregulation rather than a direct causal link.^12^

Our patient is unique as he remains alive and can be monitored for future complications related to the FA-LBCL mural thrombus. He will receive four weekly doses of rituximab, a monoclonal antibody, followed by involved-site radiation therapy. His clinical course and treatment can help guide treatment for future cases of similar diagnoses.

## Conclusion

This case highlights the challenges of diagnosing and managing FA-LBCL within a native arterial thrombus, a rare finding with only one prior reported case. The patient’s early positive outcome following surgery and initiation of rituximab therapy underscores the importance of early detection and tailored treatment. This report adds to the limited knowledge of FA-LBCL in vascular thrombus and underscores the need for further research to guide its diagnosis and management.

## Funding

None.

## Disclosures

None.
